# A Review of Adropin as the Medium of Dialogue between Energy Regulation and Immune Regulation

**DOI:** 10.1155/2020/3947806

**Published:** 2020-03-04

**Authors:** Shuyu Zhang, Qingquan Chen, Xuchen Lin, Min Chen, Qicai Liu

**Affiliations:** ^1^Department of Laboratory Medicine, Fujian Medical University, 350004 Fuzhou, China; ^2^Center for Reproductive Medicine, 1st Affiliated Hospital, Fujian Medical University, 350004 Fuzhou, China

## Abstract

Adropin is a secretory protein encoded by the energy balance gene and is closely associated with regulation of energy metabolism and insulin resistance. The clinical findings demonstrated its decreased expression in various inflammatory diseases, its negative correlation with the expression levels of inflammatory cytokines, and its potential anti-inflammatory effects. We speculate that adropin plays a pivotal regulatory role in immune cells and inflammatory factors. In this study, we reviewed the advances in researches concentrated on immunological effects of adropin.

## 1. Introduction

Adropin is a peptide hormone encoded by the energy homeostasis-associated (ENHO) gene. The nomenclature of adropin is derived from the Latin roots “aduro” and “pinquis,” meaning “promoting fat burning,” with identical amino acid sequences in humans and mice [1]. At present, the half-life of adropin has still remained elusive, and its half-life may last from several minutes to half an hour, which is similar to other secretory proteins [2]. The biological effects of adropin are mediated through activation of the orphan G protein-coupled receptor 19 (GPR19) [3, 4]. In 2008, Kumar et al. [1] found that the ENHO gene was localized on chromosome 9p13.3 in obese mice model and consisted of 25 exons. They also reported that adropin is consisted of 76 amino acids, and it was originally described as a secreted peptide, with residues 1-33 encoding a secretory signal peptide sequence. Besides, it was mainly expressed in tissues, such as liver, brain, heart, kidney, pancreas, coronary artery, and umbilical vein, and its expression was the highest in the brain. Simultaneously, the expression of ENHO gene in mouse brain by means of autoradiography and their results revealed that ENHO was highly expressed in the regions controlling complex behaviors, such as circadian rhythm and stress response. Similarly, serum adropin levels are regulated by metabolic status and diet. In the study of K. Ganesh [5], adropin levels were high in chow-fed conditions and were low in fasting, and serum adropin levels were significantly higher in mice fed a high-fat low-carbohydrate diet than in mice fed a low-fat high-carbohydrate diet. Meanwhile, diet-induced obesity (DIO) suppressed the serum adropin levels of mice. However, human serum adropin levels are not affected by acute signals such as fasting or meal, but by obesity and dietary preferences. There is a positive association between human serum adropin levels and fat intake and a negative association with carbohydrate intake [6–8].

The expression level of adropin in normal human plasma is 1-10 *μ*g/L, in which its expression level in males is slightly higher than that in females [6]. Meanwhile, the expression level of adropin is reduced with increase of age [9].

## 2. Overview on Functions of Adropin

A number of scholars studied functions of adropin, while they have mainly concentrated on metabolic disorders and cardiovascular diseases. Adropin enhances glucose oxidation and ameliorates metabolic inflexibility of utilizing glucose in obese and insulin-resistant mice. The underlying mechanisms appear to involve suppressions of carnitine palmitoyltransferase-1B (CPT-1B) and CD36, two key enzymes in fatty acid utilization. Adropin treatment activates pyruvate dehydrogenase (PDH), a rate-limiting enzyme in glucose oxidation, and downregulates PDH kinase-4 (PDK-4) that inhibits PDH [10]. Adropin can up-regulate the endothelial nitric oxide synthase (eNOS) expression through VEGFR2-PI3K-Akt or VEGFR2-ERK1/2 pathway, increase the release of NO, improve endothelial cell function, and promote the neovascularization, thereby protecting the cardiovascular system [11]. In recent years, the role of adropin in the central nervous system (CNS) has also been studied. It has been shown that adropin acts as a plasma membrane-binding protein in CNS, interacts with brain-specific Notch1 ligand NB3, regulates physical activity and motor coordination through the NB3/Notch signaling pathway, and plays a pivotal role in cerebellum development in mice [12]. It also exerts neuroprotective effects by reducing oxidative damage [9]. In studies of the association of adropin with atherosclerosis and insulin resistance, in addition to its role in regulating metabolism and improving functions of endothelial cells, the immunological effects of adropin have gradually attracted scholars' attention.

## 3. Metabolic Disorders Caused by the Immune Regulation of Adropin

Obesity intervention results from a persistent energy imbalance. Adipose tissue is increasingly considered as a key regulator of energy balance and is a “crossroad” of energy homeostasis, inflammation, and atherosclerosis [13]. If the number of free fatty acid (FFA) exceeds the storage capacity of the adipose tissue, it may overflow and may be accumulated in metabolic tissues, such as skeletal muscle, liver, and pancreas; excessive FFA can activate inflammatory pathways and damage immune system and adipose tissues, thereby leading to cell dysfunction [14, 15]. Therefore, fatty acid can regulate the function and inflammation phenotype of immune cells, playing a substantial role in causing metabolic disorders, such as insulin resistance and type 2 diabetes.

**Figure 1:**
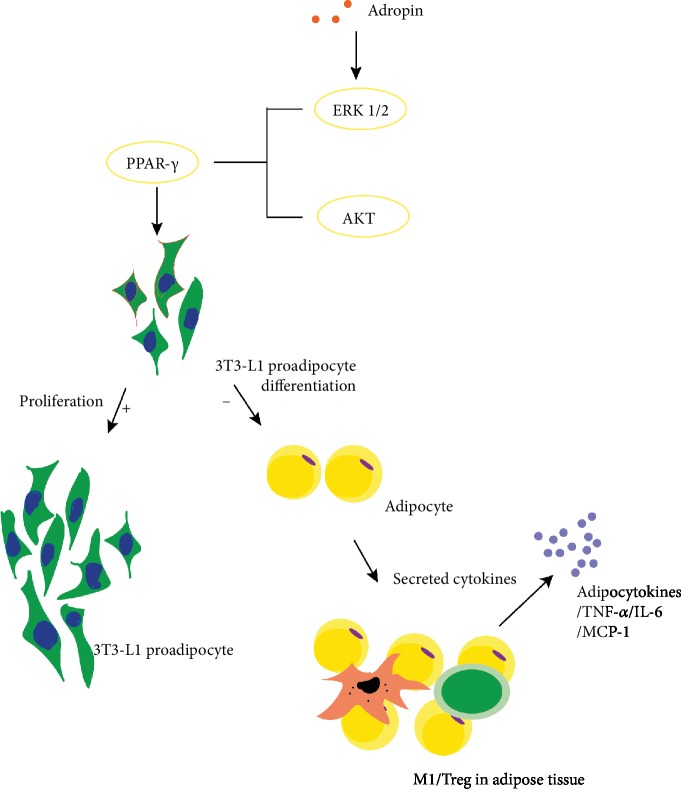
Infiltration of macrophages in adipose tissues causes chronic inflammation. Adipocytes are able to secrete cytokines such as TNF-*α* and MCP-1 that attract macrophages and Treg cells, leading to fat inflammation. Adropin regulates the expression of PPAR-*γ* by activating EK1/2 and AKT pathways, thus promoting the proliferation of 3T3-L1 preadipocytes and inhibiting the differentiation of 3T3-L1 preadipocytes into mature adipocytes and thus reducing fat accumulation and fat inflammation.

Numerous studies demonstrated that visceral adipose tissue is associated with macrophages in chronic inflammatory conditions around the adipocytes, and infiltration of visceral adipose tissues by proinflammatory macrophages is a key event driving adipose-tissue inflammation and insulin resistance [14]. The macrophages in the adipose tissue are the main source of inflammatory cytokines, such as tumor necrosis factor *α* (TNF-*α*), a multifunctional proinflammatory cytokine that plays a significant role in the inflammatory process [16, 17]. The fat content is positively correlated with the number of macrophages, and the ablation of adipose tissues leads to a decrease in systemic inflammation [18]. Adropin can regulate the expressions of lipogenic genes and peroxisome proliferator-activated receptor *γ* (PPAR-*γ*) in the adipose tissues and liver, and is a main regulator of lipogenesis as well. Besides, PPAR-*γ* was found to be significantly decreased in mice with overexpression of adropin [1]. A recently conducted study demonstrated that adropin promotes the proliferation of 3T3-L1 preadipocyte via mediating ERK1/2 and AKT (Figure 1), and inhibits differentiation of preadipocytes into mature adipocytes by reducing lipid accumulation and expressions of adipogenic genes in 3T3-L1 cells and rat preadipocytes [19]. Thus, adropin can reduce macrophage infiltration by decreasing fat accumulation, thereby improving inflammation.

Treg cells are involved in controlling the inflammatory state of adipose tissues. Treg cells are the main cells responsible for the negative regulation of immune-mediated inflammation. It is involved in the negative regulation of autoimmune diseases, allergies, acute, and chronic infections, cancer, and metabolic inflammation. In obese mice, the number of Treg cells in adipose tissue is strikingly reduced, and the imbalance of immune cells leads to fat inflammation. Meanwhile, the decrease of Treg cells in adipose tissue also leads to the occurrence of insulin resistance, so it is believed that Treg cells play an important role in metabolic regulation [20, 21]. Additionally, a previous research reported that adropin deficiency associates with loss of Treg cells and leads to autoimmune diseases [22].

**Figure 2:**
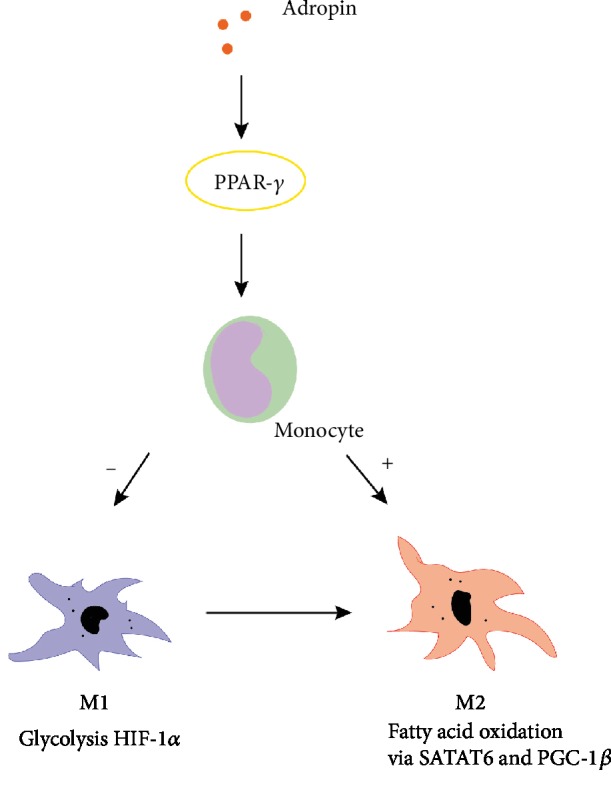
Regulatory mechanisms of adropin for immune cells. M1 macrophages use the energy provided by aerobic glycolysis for proinflammatory responses, while M2 macrophages depend on the energy provided by fatty acid oxidation for anti-inflammatory responses. Adropin regulates macrophage polarization by regulating the expression of PPAR-*γ*, a gene related to fatty acid metabolism.

PPAR-*γ* is highly expressed in adipose tissues and plays an irreplaceable role in adipocyte differentiation, and is involved in fatty acid metabolism. In addition, activation of PPAR-*γ* has potential effects on the expressions and secretions of numerous factors, including reducing expressions and secretions of adipokines, such as adiponectin and resisting, and proinflammatory cytokines (e.g., interleukin 6 (IL-6), TNF-*α*, and monocyte chemotactic protein-1 (MCP-1)); MCP-1 and TNF-*α* can induce macrophage infiltration and inflammation [23]. Therefore, activation of PPAR-*γ* may reduce macrophage infiltration and inflammation of adipose tissues. The study demonstrated that adropin regulates the anti-inflammatory or proinflammatory phenotypes of macrophages by up-regulating the expression of PPAR-*γ* [24]. Although, in current research, the reason for the tissue-specific effects of adropin on PPAR-*γ* expression is often unclear, PPAR-*γ* may be an important target for adropin to exert anti-inflammatory effects (Figure 2). Another study showed that M1 macrophages use aerobic glycolysis to provide energy for rapid, transient bactericidal effect or proinflammatory responses. Conversely, M2 macrophages depend on the energy provided by fatty acid oxidation (FAO) to exert anti-inflammatory effects for a long period of time [25]. The change in the polarization of macrophages varies according to the diversity of cytokines present in the microenvironment or by the stimuli of an antigen. It involves interferon-regulatory factors, such as PPARs, hypoxia-inducible factors (HIFs), and signal transducers and activators of transcription [26]. It also has been reported that in macrophages, PPAR-*γ* has been shown to play critical roles in inflammation and metabolism [27]. However, further research is required to indicate whether adropin can alter the macrophage phenotype by regulating cell metabolism.

Adropin plays a significant role in other metabolic disorders, such as diabetic nephropathy, polycystic ovary syndrome (PCOS), etc. Studies indicated that adropin can significantly reduce the expressions of TNF-*α*, IL-6, and inducible NOS (iNOS) at the mRNA level in pancreatic tissues of diabetic rats [28, 29]. Furthermore, decreased level of adropin is associated with an increase in the inflammatory marker (TNF-*α*) in women with PCOS [30]. The above-mentioned findings demonstrated that the expression level of adropin can be reduced in various inflammatory metabolic diseases.

## 4. Correlation between Inhibition of Inflammation by Adropin and Cardiovascular Diseases

Studies on the correlation between adropin and pathogenesis of cardiovascular diseases mainly concentrated on the protection and regulation of function of endothelial cells by adropin. Adropin can also upregulate the expression level of eNOS by upregulating PI3K/Akt and extracellular signal-regulated kinase (ERK) signal transduction pathways in vitro and in vivo, thereby increasing bioavailability of NO [11]. On the one hand, as an endogenous vasodilator, NO plays a substantial role in maintaining the homeostasis of endothelial cells [31]; on the other hand, NO can exert immunomodulatory influences in inhibiting adhesion of monocytes and leukocytes to the endothelia [32]. Sato et al. [24] demonstrated that adropin can inhibit TNF-*α*-induced adhesion of THP1 monocytes to endothelial cells in the process of atherosclerosis. With impeding monocyte-endothelial cell interactions, it can inhibit the inflammatory response of endothelial cells and monocytes/macrophages. With regulation of the phenotype of macrophages, it exerts proinflammatory or anti-inflammatory effects on atherosclerosis. In terms of energy metabolism, metabolic disorders caused by insulin resistance or inflammation leads to activations of inflammatory transcription factor nuclear factor kB (NF-*κ*B) and inflammatory signaling system, as well as elevated levels of cytokines, thereby accelerating the damage to function of endothelial cells and formation of atherosclerotic plaques [22]. As a regulator of energy metabolism, adropin may exert its potential anti-inflammatory effects through regulation of energy metabolism.

Additionally, in studies on cardiovascular diseases, such as coronary artery disease (CAD) and atherosclerosis, scholars found that adropin has a significant negative correlation with homocysteine (Hcy), hypersensitive C-reactive protein (hs-CRP), and levels of cytokines. (1) Hcy: Hcy is known to mediate cardiovascular problems by its adverse effects on cardiovascular endothelium and smooth muscle cells with resultant alterations in subclinical arterial structure and function. Serum Hcy level is negatively correlated with serum adropin level in CAD patients [33]. Hyperhomocysteinemia activates c-Jun N-terminal kinase by inducing endoplasmic reticulum stress, which can stimulate the production of proinflammatory cytokines and promote macrophage infiltration, thereby promoting insulin resistance [34]. (2) Inflammatory cytokines: studies conducted by Sato et al. [24] showed that adropin could reduce the expressions of TNF-*α* and IL-6 at the mRNA level by regulating the expression of iNOS, thereby exerting anti-inflammatory effects on the atherosclerosis. (3) CD36: CD36, which can be downregulated by adropin, is a multi-ligand and multifunctional inflammatory receptor that induces inflammatory responses through activation of various ligands and cellular responses; for example, the interaction with fibrillar *β*-amyloid (fA*β*)/integrin can induce an inflammatory response by increasing expressions of proinflammatory cytokines and chemokines; CD36 receptor for oxidized low-density lipoprotein (oxLDL) can alter cytoskeletal dynamics, enhance macrophage spreading, and inhibit migration. This may result in macrophages being captured in the endarterium, as well as further promoting atherosclerosis [10, 35, 36]. (4) hs-CRP: adropin is negatively correlated with acute inflammatory marker (hs-CRP), which can also provide strong evidence for the anti-inflammatory effect of adropin.

## 5. Association between Adropin and Other Inflammatory Diseases

In addition to metabolic disorders and cardiovascular diseases, adropin has been shown as a potential anti-inflammatory factor in other inflammatory diseases. Gao et al. [37] demonstrated that ENHO-/- mice showed MPO-ANCA-related pulmonary vasculitis, which is an autoimmune disease. It is well known that Treg cell is a subset of T cells that control autoimmune reactivity, and their deficiency can lead to autoimmune diseases. In the lung tissues of AdrKO mice, the number and ratio of Treg cells were found to be significantly reduced. At the same time, there was a sharp increase in CD3, CD20, and CD38 positive cells in the lung tissues of AdrKO mice. The neutrophil recruitment and neutrophil-endothelial cell interactions caused by ENHO mutation/adropin deficiency were associated with lung injury related to MPO-ANCA.

It was previously revealed that adropin can inhibit hepatic cell inflammation in hyperlipidemia rats [38]. In AdrKO mice, the more the accumulation of hepatic lipid, the more severe the inflammatory response, and the expressions of inflammation-related genes (Il1b, Il6, and Tnf) were remarkably elevated [39]. This may be attributed to the regulatory effects of adropin on the accumulation of hepatic lipid. However, it also suggested that in a variety of inflammations, various tissues, and even blood, the level of adropin is associated with inflammation-related genes (especially Il6 and Tnf). In patients with knee osteoarthritis, the level of adropin is negatively correlated with TNF-*α* level, white blood cell (WBC) count, and neutrophil-lymphocyte ratio (NLR) [40]. The underlying mechanism may be related to the upregulation of eNOS activity by adropin, and the produced NO can negatively regulate inflammatory mediators. Furthermore, it can impede the leukocyte extravasation and movement process regulated by TNF-*α*, thereby applying its anti-inflammatory effects [41].

**Figure 3:**
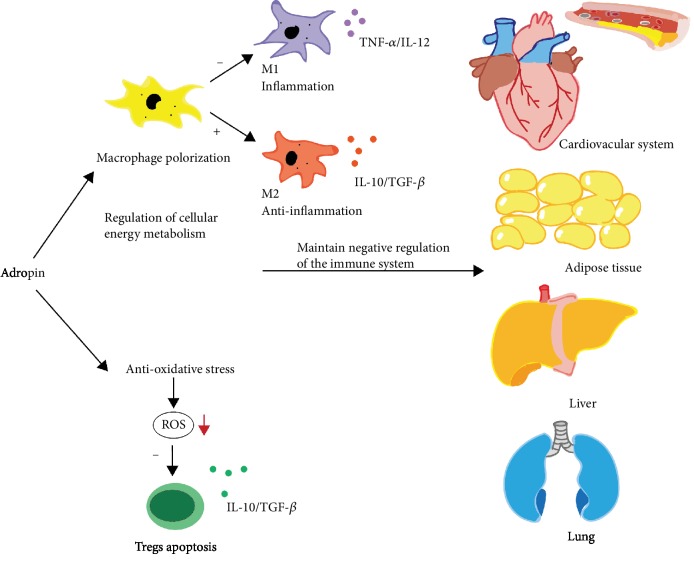
Adropin plays an anti-inflammatory role in a variety of tissues. Adropin can affect macrophage polarization by regulating cell energy metabolism and prevent ROS-induced apoptosis of Tregs through antioxidant stress. Thus, it can maintain the negative regulation of the immune system and play an anti-inflammatory role in atherosclerosis, fat inflammation, fatty liver, nonalcoholic hepatitis, and pulmonary vasculitis. Adropin deficiency can lead to imbalance of immune cells and inflammatory cytokines, which will destroy the negative regulation of the immune system and result in inflammation.

Adropin has the effect of antioxidative stress. Study has shown that adropin deficiency correlates with increased oxidative stress associated with endothelial dysfunction in the brain of rats [9]. Meanwhile, adropin can activate ERK 1/2 through VEGFR2, and ERK 1/2 activation induces Nrf2 and protect neurons from oxidative stress [42]. Inhibition of ERK 1/2 may reduce DNA repairing ability, accelerate cell apoptosis, and aggravate neuron loss [43]. The antioxidative stress effect of adropin is also related to its immune regulation function. Adropin activates Nrf2 signaling in nonalcoholic steatohepatitis (NASH) and plays a role in decreasing reactive oxygen species (ROS) production from liver mitochondria. So, it may protect mitochondrial function to alleviate oxidative stress and apoptosis and thus protect against liver injury and prevent the NASH progression [44]. Excessive reactive oxygen production can cause inflammation [45]. The study indicated that the increase of oxidative stress in a fatty liver caused the apoptosis of Tregs, reduced the number of hepatic Tregs, and led to a lowered suppression of inflammatory responses. This is because increased fatty acid metabolism leads to increased mitochondrial respiratory activity and excessive production of mitochondrial ROS in the liver, which can reduce the expression of bcl-2 in Tregs and selectively affected a subpopulation of T lymphocytes (Tregs) (Figure 3) [46, 47].

## 6. Prospect

At present, studies concentrated on adropin protein and its functions are still in the preliminary stage. However, there is increasing evidence that adropin is highly associated with various inflammatory diseases, and is also involved in the inflammatory process of different diseases. Moreover, it plays a substantial role in regulating the phenotype and biological behavior of immune cells, in addition to the secretion of inflammatory cytokines. The specific mechanisms have not been fully and systematically elucidated, and a larger sample size is also required to confirm the immunomodulatory effects of adropin. Nevertheless, as a potential anti-inflammatory protein, its immunological effects should be investigated in the future researches.
